# Supplementary material for: NLRP3 inflammasome and pyroptosis: implications in inflammation and multisystem disorders
Source: PeerJ. 2025 Aug 15;13:e19887. doi: 10.7717/peerj.19887 (PMC12360325; doi:10.7717/peerj.19887)
Supplement: Supplemental Information 1 [file peerj-13-19887-s001.docx]

**REFERENCES**

Afsar B, Afsar RE, Ertuglu LA, Covic A, Kanbay M. 2022. Nutrition, immunology, and kidney: Looking beyond the horizons. *Current Nutrition Reports* 11:69–81. DOI: 10.1007/s13668-021-00388-8.

Bachus S, Graves D, Fulham L, Akkerman N, Stephanson C, Shieh J, Pelka P. 2022. In mitosis you are not: The NIMA family of kinases in aspergillus, yeast, and mammals. *International Journal of Molecular Sciences* 23:4041. DOI: 10.3390/ijms23074041.

Balasubramanian A, Hsu AY, Ghimire L, Tahir M, Devant P, Fontana P, Du G, Liu X, Fabin D, Kambara H, Xie X, Liu F, Hasegawa T, Xu R, Yu H, Chen M, Kolakowski S, Trauger S, Larsen MR, Wei W, Wu H, Kagan JC, Lieberman J, Luo HR. 2024. The palmitoylation of gasdermin D directs its membrane translocation and pore formation during pyroptosis. *Science Immunology* 9:eadn1452. DOI: 10.1126/sciimmunol.adn1452.

Bazrafkan M, Hosseini E, Nazari M, Amorim CA, Sadeghi MR. 2021. NLRP3 inflammasome: a joint, potential therapeutic target in management of COVID-19 and fertility problems. *Journal of Reproductive Immunology* 148:103427. DOI: 10.1016/j.jri.2021.103427.

Broz P. 2023. Unconventional protein secretion by gasdermin pores. *Seminars in Immunology* 69:101811. DOI: 10.1016/j.smim.2023.101811.

Broz P, Pelegrín P, Shao F. 2020. The gasdermins, a protein family executing cell death and inflammation. *Nature Reviews. Immunology* 20:143–157. DOI: 10.1038/s41577-019-0228-2.

Cai Y, Chai Y, Fu Y, Wang Y, Zhang Y, Zhang X, Zhu L, Miao M, Yan T. 2021. Salidroside ameliorates alzheimer’s disease by targeting NLRP3 inflammasome-mediated pyroptosis. *Frontiers in Aging Neuroscience* 13:809433. DOI: 10.3389/fnagi.2021.809433.

Chen Y, Zhou C, Bian Y, Fu F, Zhu B, Zhao X, Zhang M, Zhou C, Yao S, Zhang Z, Luo H, Ge Y, Wu C, Ruan H. 2023a. Cadmium exposure promotes thyroid pyroptosis and endocrine dysfunction by inhibiting Nrf2/Keap1 signaling. *Ecotoxicology and Environmental Safety* 249:114376. DOI: 10.1016/j.ecoenv.2022.114376.

Chen J, Zhu Z, Xu S, Li J, Huang L, Tan W, Zhang Y, Zhao Y. 2023b. HDAC1 participates in polycystic ovary syndrome through histone modification by regulating H19/miR-29a-3p/NLRP3-mediated granulosa cell pyroptosis. *Molecular and Cellular Endocrinology* 573:111950. DOI: 10.1016/j.mce.2023.111950.

Cheng Z, Huang M, Li W, Hou L, Jin L, Fan Q, Zhang L, Li C, Zeng L, Yang C, Liang B, Li F, Chen C. 2024. HECTD3 inhibits NLRP3 inflammasome assembly and activation by blocking NLRP3-NEK7 interaction. *Cell Death and Disease* 15:86. DOI: 10.1038/s41419-024-06473-4.

Chou W-C, Jha S, Linhoff MW, Ting JP-Y. 2023. The NLR gene family: From discovery to present day. *Nature Reviews. Immunology* 23:635–654. DOI: 10.1038/s41577-023-00849-x.

Coll RC, Robertson AAB, Chae JJ, Higgins SC, Muñoz-Planillo R, Inserra MC, Vetter I, Dungan LS, Monks BG, Stutz A, Croker DE, Butler MS, Haneklaus M, Sutton CE, Núñez G, Latz E, Kastner DL, Mills KHG, Masters SL, Schroder K, Cooper MA, O’Neill LAJ. 2015. A small-molecule inhibitor of the NLRP3 inflammasome for the treatment of inflammatory diseases. *Nature Medicine* 21:248–255. DOI: 10.1038/nm.3806.

David L, Borges JP, Hollingsworth LR, Volchuk A, Jansen I, Garlick E, Steinberg BE, Wu H. 2024. NINJ1 mediates plasma membrane rupture by cutting and releasing membrane disks. *Cell* 187:2224-2235.e16. DOI: 10.1016/j.cell.2024.03.008.

Degen M, Santos JC, Pluhackova K, Cebrero G, Ramos S, Jankevicius G, Hartenian E, Guillerm U, Mari SA, Kohl B, Müller DJ, Schanda P, Maier T, Perez C, Sieben C, Broz P, Hiller S. 2023. Structural basis of NINJ1-mediated plasma membrane rupture in cell death. *Nature* 618:1065–1071. DOI: 10.1038/s41586-023-05991-z.

Dg P, P M, R L, N H, H P, E S, J E, R G, Ma D, Ca G. 2001. Identification and characterization of a novel class of interleukin-1 post-translational processing inhibitors. *Journal of Pharmacology and Experimental Therapeutics* 299.

Dias C, Hornung V, Nylandsted J. 2022. A novel NINJ1-mediated regulatory step is essential for active membrane rupture and common to different cell death pathways. *Faculty Reviews* 11:41. DOI: 10.12703/r-01-0000021.

Ding J, Wang K, Liu W, She Y, Sun Q, Shi J, Sun H, Wang D-C, Shao F. 2016. Pore-forming activity and structural autoinhibition of the gasdermin family. *Nature* 535:111–116. DOI: 10.1038/nature18590.

de Dios C, Abadin X, Roca-Agujetas V, Jimenez-Martinez M, Morales A, Trullas R, Mari M, Colell A. 2023. Inflammasome activation under high cholesterol load triggers a protective microglial phenotype while promoting neuronal pyroptosis. *Translational Neurodegeneration* 12:10. DOI: 10.1186/s40035-023-00343-3.

D’Souza CA, Heitman J. 2001. Dismantling the cryptococcus coat. *Trends in Microbiology* 9:112–113. DOI: 10.1016/s0966-842x(00)01945-4.

Du G, Healy LB, David L, Walker C, Fontana P, Dong Y, Devant P, Puthenveetil R, Ficarro SB, Banerjee A, Kagan JC, Lieberman J, Wu H. 2023. ROS-dependent palmitoylation is an obligate licensing modification for GSDMD pore formation. *Biorxiv: The Preprint Server for Biology*:2023.3.7.531538. DOI: 10.1101/2023.03.07.531538.

Duan F, Li L, Liu S, Tao J, Gu Y, Li H, Yi X, Gong J, You D, Feng Z, Yu T, Tan H. 2024. Cortistatin protects against septic cardiomyopathy by inhibiting cardiomyocyte pyroptosis through the SSTR2-AMPK-NLRP3 pathway. *International Immunopharmacology* 134:112186. DOI: 10.1016/j.intimp.2024.112186.

Fan X, Cheng Z, Shao R, Ye K, Chen X, Cai X, Dai S, Tang Z, Shi S, Zheng W, Huang W, Han J, Ye B. 2025. The novel GSDMD inhibitor GI-Y2 exerts antipyroptotic effects to reduce atherosclerosis. *Clinical and Translational Medicine* 15:e70263. DOI: 10.1002/ctm2.70263.

Foley JF. 2024. Palmitoylation promotes pores. *Science Signaling* 17:eadr1306. DOI: 10.1126/scisignal.adr1306.

Fu J, Wu H. 2023. Structural mechanisms of NLRP3 inflammasome assembly and activation. *Annual Review of Immunology* 41:301–316. DOI: 10.1146/annurev-immunol-081022-021207.

Gao C, Wang B, Chen Q, Wang M, Fei X, Zhao N. 2021. Serum exosomes from diabetic kidney disease patients promote pyroptosis and oxidative stress through the miR-4449/HIC1 pathway. *Nutrition & Diabetes* 11:33. DOI: 10.1038/s41387-021-00175-y.

Ghiringhelli F, Apetoh L, Tesniere A, Aymeric L, Ma Y, Ortiz C, Vermaelen K, Panaretakis T, Mignot G, Ullrich E, Perfettini J-L, Schlemmer F, Tasdemir E, Uhl M, Génin P, Civas A, Ryffel B, Kanellopoulos J, Tschopp J, André F, Lidereau R, McLaughlin NM, Haynes NM, Smyth MJ, Kroemer G, Zitvogel L. 2009. Activation of the NLRP3 inflammasome in dendritic cells induces IL-1beta-dependent adaptive immunity against tumors. *Nature Medicine* 15:1170–1178. DOI: 10.1038/nm.2028.

Hao W, Zhu X, Liu Z, Song Y, Wu S, Lu X, Yang J, Jin C. 2023. Aluminum exposure induces central nervous system impairment via activating NLRP3-medicated pyroptosis pathway. *Ecotoxicology and Environmental Safety* 264:115401. DOI: 10.1016/j.ecoenv.2023.115401.

Harrison D. 2024. Brain penetrant NLRP3 inhibitors: The discovery of a panacea? *Journal of Medicinal Chemistry* 67:20776–20779. DOI: 10.1021/acs.jmedchem.4c02846.

He Y, Zeng MY, Yang D, Motro B, Núñez G. 2016. NEK7 is an essential mediator of NLRP3 activation downstream of potassium efflux. *Nature* 530:354–357. DOI: 10.1038/nature16959.

Hochheiser IV, Pilsl M, Hagelueken G, Moecking J, Marleaux M, Brinkschulte R, Latz E, Engel C, Geyer M. 2022. Structure of the NLRP3 decamer bound to the cytokine release inhibitor CRID3. *Nature* 604:184–189. DOI: 10.1038/s41586-022-04467-w.

Hodel AW, Rudd-Schmidt JA, Trapani JA, Voskoboinik I, Hoogenboom BW. 2021. Lipid specificity of the immune effector perforin. *Faraday Discussions* 232:236–255. DOI: 10.1039/d0fd00043d.

Imre G. 2024. Pyroptosis in health and disease. *American Journal of Physiology. Cell Physiology* 326:C784–C794. DOI: 10.1152/ajpcell.00503.2023.

Jiang L, Wang Z, Xu T, Zhang L. 2024a. When pyro(ptosis) meets palm(itoylation). *Cytokine & Growth Factor Reviews* 77:30–38. DOI: 10.1016/j.cytogfr.2024.03.001.

Jiang X, Zhang X, Cai X, Li N, Zheng H, Tang M, Zhu J, Su K, Zhang R, Ye N, Peng J, Zhao M, Wu W, Yang J, Ye H. 2024b. NU6300 covalently reacts with cysteine-191 of gasdermin D to block its cleavage and palmitoylation. *Science Advances* 10:eadi9284. DOI: 10.1126/sciadv.adi9284.

Jiang S, Zhang H, Li X, Yi B, Huang L, Hu Z, Li A, Du J, Li Y, Zhang W. 2021. Vitamin D/VDR attenuate cisplatin-induced AKI by down-regulating NLRP3/caspase-1/GSDMD pyroptosis pathway. *Journal of Steroid Biochemistry and Molecular Biology* 206:105789. DOI: 10.1016/j.jsbmb.2020.105789.

Kang J-Y, Choi H, Oh J-M, Kim M, Lee D-C. 2024. PM2.5 induces pyroptosis via activation of the ROS/NF-κB signaling pathway in bronchial epithelial cells. *Medicina (kaunas, Lithuania)* 60:1434. DOI: 10.3390/medicina60091434.

Kanneganti T-D. 2020. Intracellular innate immune receptors: Life inside the cell. *Immunological Reviews* 297:5–12. DOI: 10.1111/imr.12912.

Kayagaki N, Kornfeld OS, Lee BL, Stowe IB, O’Rourke K, Li Q, Sandoval W, Yan D, Kang J, Xu M, Zhang J, Lee WP, McKenzie BS, Ulas G, Payandeh J, Roose-Girma M, Modrusan Z, Reja R, Sagolla M, Webster JD, Cho V, Andrews TD, Morris LX, Miosge LA, Goodnow CC, Bertram EM, Dixit VM. 2021. NINJ1 mediates plasma membrane rupture during lytic cell death. *Nature* 591:131–136. DOI: 10.1038/s41586-021-03218-7.

Keane S, Herring M, Rolny P, Wettergren Y, Ejeskär K. 2022. Inflammation suppresses DLG2 expression decreasing inflammasome formation. *Journal of Cancer Research and Clinical Oncology* 148:2295–2311. DOI: 10.1007/s00432-022-04029-7.

Klughammer B, Piali L, Nica A, Nagel S, Bailey L, Jochum C, Ignatenko S, Bläuer A, Danilin S, Gulati P, Hayward J, Scepanovic P, Zhang JD, Bhosale S, Chong CF, Christ A. 2023. A randomized, double-blind phase 1b study evaluating the safety, tolerability, pharmacokinetics and pharmacodynamics of the NLRP3 inhibitor selnoflast in patients with moderate to severe active ulcerative colitis. *Clinical and Translational Medicine* 13:e1471. DOI: 10.1002/ctm2.1471.

Lalor SJ, Dungan LS, Sutton CE, Basdeo SA, Fletcher JM, Mills KHG. 2011. Caspase-1-processed cytokines IL-1beta and IL-18 promote IL-17 production by gammadelta and CD4 T cells that mediate autoimmunity. *Journal of Immunology (baltimore, Md.: 1950)* 186:5738–5748. DOI: 10.4049/jimmunol.1003597.

Li X, Bai C, Wang H, Wan T, Li Y. 2022a. LncRNA MEG3 regulates autophagy and pyroptosis via FOXO1 in pancreatic β-cells. *Cellular Signalling* 92:110247. DOI: 10.1016/j.cellsig.2022.110247.

Li A, Gu L, He C, Li Y, Peng M, Liao J, Xiao R, Xu L, Guo S. 2023. GATA6 promotes fibrotic repair of tracheal injury through NLRP3 inflammasome-mediated epithelial pyroptosis. *International Immunopharmacology* 123:110657. DOI: 10.1016/j.intimp.2023.110657.

Li F, Liu Z. 2024. Expression of NLRP3 in serum and induced sputum of children with asthma and their relationship with disease severity. *European Journal of Medical Research* 29:526. DOI: 10.1186/s40001-024-02114-w.

Li T, Sun H, Li Y, Su L, Jiang J, Liu Y, Jiang N, Huang R, Zhang J, Peng Z. 2022b. Downregulation of macrophage migration inhibitory factor attenuates NLRP3 inflammasome mediated pyroptosis in sepsis-induced AKI. *Cell Death Discovery* 8:61. DOI: 10.1038/s41420-022-00859-z.

Liu S, Bi Y, Han T, Li YE, Wang Q, Wu NN, Xu C, Ge J, Hu R, Zhang Y. 2024a. The E3 ubiquitin ligase MARCH2 protects against myocardial ischemia-reperfusion injury through inhibiting pyroptosis via negative regulation of PGAM5/MAVS/NLRP3 axis. *Cell Discovery* 10:24. DOI: 10.1038/s41421-023-00622-3.

Liu G, Chen X, Wang Q, Yuan L. 2020. NEK7: A potential therapy target for NLRP3-related diseases. *Bioscience Trends* 14:74–82. DOI: 10.5582/bst.2020.01029.

Liu J, Chen Y, Zhang J, Zheng Y, An Y, Xia C, Chen Y, Huang S, Hou S, Deng D. 2025. Vitexin alleviates MNNG-induced chronic atrophic gastritis via inhibiting NLRP3 inflammasome. *Journal of Ethnopharmacology* 340:119272. DOI: 10.1016/j.jep.2024.119272.

Liu S, Li S, Dong Y, Qiao K, Zhao Y, Yu J. 2024b. Hispidulin targets PTGS2 to improve cyclophosphamide-induced cystitis by suppressing NLRP3 inflammasome. *Naunyn-schmiedeberg’s Archives of Pharmacology* 397:5819–5830. DOI: 10.1007/s00210-024-02987-y.

Liu W, Yang J, Fang S, Jiao C, Gao J, Zhang A, Wu T, Tan R, Xu Q, Guo W. 2022a. Spirodalesol analog 8A inhibits NLRP3 inflammasome activation and attenuates inflammatory disease by directly targeting adaptor protein ASC. *Journal of Biological Chemistry* 298:102696. DOI: 10.1016/j.jbc.2022.102696.

Liu X, Zhang Z, Ruan J, Pan Y, Magupalli VG, Wu H, Lieberman J. 2016. Inflammasome-activated gasdermin D causes pyroptosis by forming membrane pores. *Nature* 535:153–158. DOI: 10.1038/nature18629.

Liu Q, Zhao C, Zhou J, Zhang H, Zhang Y, Wang S, Pu Y, Yin L. 2022b. Reactive oxygen species-mediated activation of NLRP3 inflammasome associated with pyroptosis in het-1A cells induced by the co-exposure of nitrosamines. *Journal of Applied Toxicology: Jat* 42:1651–1661. DOI: 10.1002/jat.4332.

Long J, Sun Y, Liu S, Yang S, Chen C, Zhang Z, Chu S, Yang Y, Pei G, Lin M, Yan Q, Yao J, Lin Y, Yi F, Meng L, Tan Y, Ai Q, Chen N. 2023. Targeting pyroptosis as a preventive and therapeutic approach for stroke. *Cell Death Discovery* 9:155. DOI: 10.1038/s41420-023-01440-y.

Luksch H, Winkler S, Heymann MC, Schulze F, Hofmann SR, Roesler J, Rösen-Wolff A. 2015. Current knowledge on procaspase-1 variants with reduced or abrogated enzymatic activity in autoinflammatory disease. *Current Rheumatology Reports* 17:45. DOI: 10.1007/s11926-015-0520-5.

Luo L, Liu M, Fan Y, Zhang J, Liu L, Li Y, Zhang Q, Xie H, Jiang C, Wu J, Xiao X, Wu Y. 2022. Intermittent theta-burst stimulation improves motor function by inhibiting neuronal pyroptosis and regulating microglial polarization via TLR4/NFκB/NLRP3 signaling pathway in cerebral ischemic mice. *Journal of Neuroinflammation* 19:141. DOI: 10.1186/s12974-022-02501-2.

Madurka I, Vishnevsky A, Soriano JB, Gans SJ, Ore DJS, Rendon A, Ulrik CS, Bhatnagar S, Krishnamurthy S, Mc Harry K, Welte T, Fernandez AA, Mehes B, Meiser K, Gatlik E, Sommer U, Junge G, Rezende E, Study group. 2023. DFV890: A new oral NLRP3 inhibitor-tested in an early phase 2a randomised clinical trial in patients with COVID-19 pneumonia and impaired respiratory function. *Infection* 51:641–654. DOI: 10.1007/s15010-022-01904-w.

Martinon F, Burns K, Tschopp J. 2002. The inflammasome: A molecular platform triggering activation of inflammatory caspases and processing of proIL-beta. *Molecular Cell* 10:417–426. DOI: 10.1016/s1097-2765(02)00599-3.

Mekni N, De Rosa M, Cipollina C, Gulotta MR, De Simone G, Lombino J, Padova A, Perricone U. 2019. In silico insights towards the identification of NLRP3 druggable hot spots. *International Journal of Molecular Sciences* 20:4974. DOI: 10.3390/ijms20204974.

Meng L, Lin H, Huang X, Weng J, Peng F, Wu S. 2022. METTL14 suppresses pyroptosis and diabetic cardiomyopathy by downregulating TINCR lncRNA. *Cell Death and Disease* 13:38. DOI: 10.1038/s41419-021-04484-z.

Newton K, Strasser A, Kayagaki N, Dixit VM. 2024. Cell death. *Cell* 187:235–256. DOI: 10.1016/j.cell.2023.11.044.

Niu T, De Rosny C, Chautard S, Rey A, Patoli D, Groslambert M, Cosson C, Lagrange B, Zhang Z, Visvikis O, Hacot S, Hologne M, Walker O, Wong J, Wang P, Ricci R, Henry T, Boyer L, Petrilli V, Py BF. 2021. NLRP3 phosphorylation in its LRR domain critically regulates inflammasome assembly. *Nature Communications* 12:5862. DOI: 10.1038/s41467-021-26142-w.

Ns S, Ia H, W K, Aak AS, Ag A, Am A, L A-A. 2025. Exploring the therapeutic potential of NLRP3 inhibitors in parkinson’s disease: A systematic review of in-vivo studies. *Inflammopharmacology*. DOI: 10.1007/s10787-025-01733-x.

Pandey A, Shen C, Feng S, Man SM. 2021. Cell biology of inflammasome activation. *Trends in Cell Biology* 31:924–939. DOI: 10.1016/j.tcb.2021.06.010.

Parmar DV, Kansagra KA, Momin T, Patel HB, Jansari GA, Bhavsar J, Shah C, Patel JM, Ghoghari A, Barot A, Sharma B, Viswanathan K, Patel HV, Jain MR. 2023. Safety, tolerability, pharmacokinetics, and pharmacodynamics of the oral NLRP3 inflammasome inhibitor ZYIL1: First-in-human phase 1 studies (single ascending dose and multiple ascending dose). *Clinical Pharmacology in Drug Development* 12:202–211. DOI: 10.1002/cpdd.1162.

Ramos S, Hartenian E, Santos JC, Walch P, Broz P. 2024. NINJ1 induces plasma membrane rupture and release of damage-associated molecular pattern molecules during ferroptosis. *Embo Journal* 43:1164–1186. DOI: 10.1038/s44318-024-00055-y.

Roshanravan N, Alamdari NM, Jafarabadi MA, Mohammadi A, Shabestari BR, Nasirzadeh N, Asghari S, Mansoori B, Akbarzadeh M, Ghavami A, Ghaffari S, Ostadrahimi A. 2020. Effects of oral butyrate and inulin supplementation on inflammation-induced pyroptosis pathway in type 2 diabetes: A randomized, double-blind, placebo-controlled trial. *Cytokine* 131:155101. DOI: 10.1016/j.cyto.2020.155101.

Rui W, Xiao H, Fan Y, Ma Z, Xiao M, Li S, Shi J. 2021. Systemic inflammasome activation and pyroptosis associate with the progression of amnestic mild cognitive impairment and alzheimer’s disease. *Journal of Neuroinflammation* 18:280. DOI: 10.1186/s12974-021-02329-2.

Saad HM, Atef E, Elsayed AE. 2025. New insights on the potential role of pyroptosis in parkinson’s neuropathology and therapeutic targeting of NLRP3 inflammasome with recent advances in nanoparticle-based miRNA therapeutics. *Molecular Neurobiology*. DOI: 10.1007/s12035-025-04818-4.

Saeedi-Boroujeni A, Nashibi R, Ghadiri AA, Nakajima M, Salmanzadeh S, Mahmoudian-Sani M-R, Hanafi MG, Sharhani A, Khodadadi A. 2022. Tranilast as an adjunctive therapy in hospitalized patients with severe COVID- 19: a randomized controlled trial. *Archives of Medical Research* 53:368–377. DOI: 10.1016/j.arcmed.2022.03.002.

Schaefer SL, Hummer G. 2022. Sublytic gasdermin-D pores captured in atomistic molecular simulations. *Elife* 11:e81432. DOI: 10.7554/eLife.81432.

Shi H, Gao Y, Dong Z, Yang J, Gao R, Li X, Zhang S, Ma L, Sun X, Wang Z, Zhang F, Hu K, Sun A, Ge J. 2021. GSDMD-mediated cardiomyocyte pyroptosis promotes myocardial I/R injury. *Circulation Research* 129:383–396. DOI: 10.1161/CIRCRESAHA.120.318629.

Shi H, Wang Y, Li X, Zhan X, Tang M, Fina M, Su L, Pratt D, Bu CH, Hildebrand S, Lyon S, Scott L, Quan J, Sun Q, Russell J, Arnett S, Jurek P, Chen D, Kravchenko VV, Mathison JC, Moresco EMY, Monson NL, Ulevitch RJ, Beutler B. 2016. NLRP3 activation and mitosis are mutually exclusive events coordinated by NEK7, a new inflammasome component. *Nature Immunology* 17:250–258. DOI: 10.1038/ni.3333.

Shi J, Zhao Y, Wang K, Shi X, Wang Y, Huang H, Zhuang Y, Cai T, Wang F, Shao F. 2015. Cleavage of GSDMD by inflammatory caspases determines pyroptotic cell death. *Nature* 526:660–665. DOI: 10.1038/nature15514.

Song Y, Guo F, Zhao Y-Y, Ma X-J, Wu L-N, Yu J-F, Ji H-F, Shao M-W, Huang F-J, Zhao L, Fan X-J, Xu Y-N, Wang Q-Z, Qin G-J. 2023. Novel lncRNA-prader willi/angelman region RNA, SNRPN neighbour (PWARSN) aggravates tubular epithelial cell pyroptosis by regulating TXNIP via dual way in diabetic kidney disease. *Cell Proliferation* 56:e13349. DOI: 10.1111/cpr.13349.

Song M, Wang J, Sun Y, Pang J, Li X, Liu Y, Zhou Y, Yang P, Fan T, Liu Y, Li Z, Qi X, Li B, Zhang X, Wang J, Wang C. 2022. Inhibition of gasdermin D-dependent pyroptosis attenuates the progression of silica-induced pulmonary inflammation and fibrosis. *Acta Pharmaceutica Sinica. B* 12:1213–1224. DOI: 10.1016/j.apsb.2021.10.006.

Stine L, Humphries F. 2024. Gasdermin D palmitoylation: To cleave or not to cleave? *Trends in Immunology* 45:403–405. DOI: 10.1016/j.it.2024.05.001.

Sun J, Gan L, Lv S, Wang T, Dai C, Sun J. 2023. Exposure to di-(2-ethylhexyl) phthalate drives ovarian dysfunction by inducing granulosa cell pyroptosis via the SLC39A5/NF-κB/NLRP3 axis. *Ecotoxicology and Environmental Safety* 252:114625. DOI: 10.1016/j.ecoenv.2023.114625.

Sun Z, Hornung V. 2024. A critical role for palmitoylation in pyroptosis. *Molecular Cell* 84:2218–2220. DOI: 10.1016/j.molcel.2024.05.023.

Tajima T, Yoshifuji A, Matsui A, Itoh T, Uchiyama K, Kanda T, Tokuyama H, Wakino S, Itoh H. 2019. β-hydroxybutyrate attenuates renal ischemia-reperfusion injury through its anti-pyroptotic effects. *Kidney International* 95:1120–1137. DOI: 10.1016/j.kint.2018.11.034.

Tang F, Kunder R, Chu T, Hains A, Nguyen A, McBride JM, Zhong Y, Santagostino S, Wilson M, Trenchak A, Chen L, Ly J, Moein A, Lewin-Koh N, Raghavan V, Osaghae U, Wynne C, Owen R, Place D. 2023. First-in-human phase 1 trial evaluating safety, pharmacokinetics, and pharmacodynamics of NLRP3 inflammasome inhibitor, GDC-2394, in healthy volunteers. *Clinical and Translational Science* 16:1653–1666. DOI: 10.1111/cts.13576.

Volchuk A, Ye A, Chi L, Steinberg BE, Goldenberg NM. 2020. Indirect regulation of HMGB1 release by gasdermin D. *Nature Communications* 11:4561. DOI: 10.1038/s41467-020-18443-3.

Wang Y, Ding L, Wang R, Guo Y, Yang Z, Yu L, Wang L, Liang Y, Tang L. 2022a. Circ_0004951 promotes pyroptosis of renal tubular cells via the NLRP3 inflammasome in diabetic kidney disease. *Frontiers in Medicine* 9:828240. DOI: 10.3389/fmed.2022.828240.

Wang JN, Li HB, Dong XW, Wu WD, Ren WJ, Yao SQ. 2022b. [role of pyroptosis pathway related molecules in acute lung injury induced by gas explosion in rats]. *Zhonghua Lao Dong Wei Sheng Zhi Ye Bing Za Zhi = Zhonghua Laodong Weisheng Zhiyebing Zazhi = Chinese Journal of Industrial Hygiene and Occupational Diseases* 40:97–102. DOI: 10.3760/cma.j.cn121094-20201009-00561.

Wang Y, Li Q, Zhao J, Chen J, Wu D, Zheng Y, Wu J, Liu J, Lu J, Zhang J, Wu Z. 2023. Mechanically induced pyroptosis enhances cardiosphere oxidative stress resistance and metabolism for myocardial infarction therapy. *Nature Communications* 14:6148. DOI: 10.1038/s41467-023-41700-0.

Wang L, Li J, Zhu Y, Zha B. 2022c. Low tidal volume ventilation alleviates ventilator-induced lung injury by regulating the NLRP3 inflammasome. *Experimental Lung Research* 48:168–177. DOI: 10.1080/01902148.2022.2104409.

Wang B, Shi M, Yu C, Pan H, Shen H, Du Y, Zhang Y, Liu B, Xi D, Sheng J, Huang H, Ding G. 2024. NLRP3 inflammasome-dependent pathway is involved in the pathogenesis of polycystic ovary syndrome. *Reproductive Sciences (thousand Oaks, Calif.)* 31:1017–1027. DOI: 10.1007/s43032-023-01348-z.

Wei S, Feng M, Zhang S. 2022. Molecular characteristics of cell pyroptosis and its inhibitors: A review of activation, regulation, and inhibitors. *International Journal of Molecular Sciences* 23:16115. DOI: 10.3390/ijms232416115.

Wei Y, You Y, Zhang J, Ban J, Min H, Li C, Chen J. 2023. Crystalline silica-induced macrophage pyroptosis interacting with mitophagy contributes to pulmonary fibrosis via modulating mitochondria homeostasis. *Journal of Hazardous Materials* 454:131562. DOI: 10.1016/j.jhazmat.2023.131562.

Wen S, Li S, Li L, Fan Q. 2020. circACTR2: A novel mechanism regulating high glucose-induced fibrosis in renal tubular cells via pyroptosis. *Biological & Pharmaceutical Bulletin* 43:558–564. DOI: 10.1248/bpb.b19-00901.

Wu D-M, He M, Zhao Y-Y, Deng S-H, Liu T, Zhang T, Zhang F, Wang Y-Y, Xu Y. 2022. Increased susceptibility of irradiated mice to aspergillus fumigatus infection via NLRP3/GSDMD pathway in pulmonary bronchial epithelia. *Cell Communication and Signaling: Ccs* 20:98. DOI: 10.1186/s12964-022-00907-2.

Xiao C, Ghosh S. 2005. NF-kappaB, an evolutionarily conserved mediator of immune and inflammatory responses. *Advances in Experimental Medicine and Biology* 560:41–45. DOI: 10.1007/0-387-24180-9_5.

Xie S, Song S, Liu S, Li Q, Zou W, Ke J, Wang C. 2024. (pro)renin receptor mediates tubular epithelial cell pyroptosis in diabetic kidney disease via DPP4-JNK pathway. *Journal of Translational Medicine* 22:26. DOI: 10.1186/s12967-023-04846-5.

Xu X-X, Shi R-X, Fu Y, Wang J-L, Tong X, Zhang S-Q, Wang N, Li M-X, Tong Y, Wang W, He M, Liu B-Y, Chen G-L, Guo F. 2023. Neuronal nitric oxide synthase/reactive oxygen species pathway is involved in apoptosis and pyroptosis in epilepsy. *Neural Regeneration Research* 18:1277–1285. DOI: 10.4103/1673-5374.357906.

Xu J, Zhang L, Duan Y, Sun F, Odeh N, He Y, Núñez G. 2025. NEK7 phosphorylation amplifies NLRP3 inflammasome activation downstream of potassium efflux and gasdermin D. *Science Immunology* 10:eadl2993. DOI: 10.1126/sciimmunol.adl2993.

Xue J, Zhou Z, Zhu Z, Sun Q, Zhu Y, Wu P. 2024. A high salt diet impairs the bladder epithelial barrier and activates the NLRP3 and NF‑κB signaling pathways to induce an overactive bladder in vivo. *Experimental and Therapeutic Medicine* 28:362. DOI: 10.3892/etm.2024.12651.

Xx Z, Xy M, Ay Z, Cy Z, C C, Tx C, Yb H, Jp X, X F, Ww C, B H, B D, Gl Z, Jr Z, Qb L, N B, Zj H, N B, Ly Q, Hj S. 2024. Vaccarin alleviates septic cardiomyopathy by potentiating NLRP3 palmitoylation and inactivation. *Phytomedicine : International Journal of Phytotherapy and Phytopharmacology* 131. DOI: 10.1016/j.phymed.2024.155771.

Yan M, Li Y, Luo Q, Zeng W, Shao X, Li L, Wang Q, Wang D, Zhang Y, Diao H, Rong X, Bai Y, Guo J. 2022. Mitochondrial damage and activation of the cytosolic DNA sensor cGAS-STING pathway lead to cardiac pyroptosis and hypertrophy in diabetic cardiomyopathy mice. *Cell Death Discovery* 8:258. DOI: 10.1038/s41420-022-01046-w.

Yang S, Cao J, Wang Y, Chen Q, Li F, Gao Y, Li R, Yuan L. 2024. Small intestinal endocrine cell derived exosomal ACE2 protects islet β-cell function by inhibiting the activation of NLRP3 inflammasome and reducing β-cell pyroptosis. *International Journal of Nanomedicine* 19:4957–4976. DOI: 10.2147/IJN.S450337.

Yang X, Chen Z, Luo Z, Yang D, Hao Y, Hu J, Feng J, Zhu Z, Luo Q, Zhang Z, Liang W, Ding G. 2023. STING deletion alleviates podocyte injury through suppressing inflammation by targeting NLRP3 in diabetic kidney disease. *Cellular Signalling* 109:110777. DOI: 10.1016/j.cellsig.2023.110777.

Yang W, Ma Y, Wu Y, Lei X, Zhang J, Li M. 2025. Study on the effects of mogroside V in inhibiting NLRP3-mediated granulosa cell pyroptosis and insulin resistance to improve PCOS. *Journal of Ovarian Research* 18:10. DOI: 10.1186/s13048-024-01563-5.

Yin M, Marrone L, Peace CG, O’Neill LAJ. 2023. NLRP3, the inflammasome and COVID-19 infection. *Qjm: Monthly Journal of The Association Of Physicians* 116:502–507. DOI: 10.1093/qjmed/hcad011.

Yu S, Yin J-J, Miao J-X, Li S-G, Huang C-Z, Huang N, Fan T-L, Li X-N, Wang Y-H, Han S-N, Zhang L-R. 2020. Activation of NLRP3 inflammasome promotes the proliferation and migration of esophageal squamous cell carcinoma. *Oncology Reports* 43:1113–1124. DOI: 10.3892/or.2020.7493.

Yuan Q, Sun Y, Yang F, Yan D, Shen M, Jin Z, Zhan L, Liu G, Yang L, Zhou Q, Yu Z, Zhou X, Yu Y, Xu Y, Wu Q, Luo J, Hu X, Zhang C. 2023. CircRNA DICAR as a novel endogenous regulator for diabetic cardiomyopathy and diabetic pyroptosis of cardiomyocytes. *Signal Transduction and Targeted Therapy* 8:99. DOI: 10.1038/s41392-022-01306-2.

Yuan C, Yu C, Sun Q, Xiong M, Ren B, Zhong M, Peng Q, Zeng M, Meng P, Li L, Song H. 2024. Atractylenolide I alleviates indomethacin-induced gastric ulcers in rats by inhibiting NLRP3 inflammasome activation. *Journal of Agricultural and Food Chemistry* 72:14165–14176. DOI: 10.1021/acs.jafc.3c08188.

Zaki MH, Boyd KL, Vogel P, Kastan MB, Lamkanfi M, Kanneganti T-D. 2010. The NLRP3 inflammasome protects against loss of epithelial integrity and mortality during experimental colitis. *Immunity* 32:379–391. DOI: 10.1016/j.immuni.2010.03.003.

Zeng C, Duan F, Hu J, Luo B, Huang B, Lou X, Sun X, Li H, Zhang X, Yin S, Tan H. 2020. NLRP3 inflammasome-mediated pyroptosis contributes to the pathogenesis of non-ischemic dilated cardiomyopathy. *Redox Biology* 34:101523. DOI: 10.1016/j.redox.2020.101523.

Zhang Z, Hu H, Luo Q, Yang K, Zou Z, Shi M, Liang W. 2024a. Dihydroxyacetone phosphate accumulation leads to podocyte pyroptosis in diabetic kidney disease. *Journal of Cellular and Molecular Medicine* 28:e18073. DOI: 10.1111/jcmm.18073.

Zhang X, Huang X, Hang D, Jin J, Li S, Zhu Y, Liu H. 2024b. Targeting pyroptosis with nanoparticles to alleviate neuroinflammatory for preventing secondary damage following traumatic brain injury. *Science Advances* 10:eadj4260. DOI: 10.1126/sciadv.adj4260.

Zhang H, Song L, Zhou L, Li X, Xuan M, Liu C, Zhao H. 2025. α -Lipoic acid alleviates Parkinson’s disease by suppressing S100A9-mediated pyroptosis. *International Immunopharmacology* 155:114539. DOI: 10.1016/j.intimp.2025.114539.

Zhang T, Tian J, Fan J, Liu X, Wang R. 2023. Exercise training-attenuated insulin resistance and liver injury in elderly pre-diabetic patients correlates with NLRP3 inflammasome. *Frontiers in Immunology* 14:1082050. DOI: 10.3389/fimmu.2023.1082050.

Zhang N, Zhang J, Yang Y, Shan H, Hou S, Fang H, Ma M, Chen Z, Tan L, Xu D. 2024c. A palmitoylation-depalmitoylation relay spatiotemporally controls GSDMD activation in pyroptosis. *Nature Cell Biology* 26:757–769. DOI: 10.1038/s41556-024-01397-9.

Zhao N, Di B, Xu L. 2021. The NLRP3 inflammasome and COVID-19: Activation, pathogenesis and therapeutic strategies. *Cytokine & Growth Factor Reviews* 61:2–15. DOI: 10.1016/j.cytogfr.2021.06.002.

Zhao N, Li C-C, Di B, Xu L-L. 2020. Recent advances in the NEK7-licensed NLRP3 inflammasome activation: Mechanisms, role in diseases and related inhibitors. *Journal of Autoimmunity* 113:102515. DOI: 10.1016/j.jaut.2020.102515.

Zheng Y, Zhang X, Wang Z, Zhang R, Wei H, Yan X, Jiang X, Yang L. 2024. MCC950 as a promising candidate for blocking NLRP3 inflammasome activation: A review of preclinical research and future directions. *Archiv Der Pharmazie* 357:e2400459. DOI: 10.1002/ardp.202400459.

Zhong L, Han J, Fan X, Huang Z, Su L, Cai X, Lin S, Chen X, Huang W, Dai S, Ye B. 2023. Novel GSDMD inhibitor GI-Y1 protects heart against pyroptosis and ischemia/reperfusion injury by blocking pyroptotic pore formation. *Basic Research in Cardiology* 118:40. DOI: 10.1007/s00395-023-01010-4.

Zhu X, Lin X, Zhang P, Liu Y, Ling W, Guo H. 2022. Upregulated NLRP3 inflammasome activation is attenuated by anthocyanins in patients with nonalcoholic fatty liver disease: A case-control and an intervention study. *Clinics and Research in Hepatology And Gastroenterology* 46:101843. DOI: 10.1016/j.clinre.2021.101843.

E G, B M, E V, U S, E T, G L, X L, P P, M V, Ws D, Y F, A O, D D, G J. 2024. First-in-human safety, tolerability, and pharmacokinetic results of DFV890, an oral low-molecular-weight NLRP3 inhibitor. *Clinical and Translational Science* 17:e13789. DOI: 10.1111/cts.13789.

Hosseini E, Kohan-Ghadr H-R, Bazrafkan M, Amorim CA, Askari M, Zakeri A, Mousavi SN, Kafaeinezhad R, Afradiasbagharani P, Esfandyari S, Nazari M. 2023. Rescuing fertility during COVID-19 infection: Exploring potential pharmacological and natural therapeutic approaches for comorbidity, by focusing on NLRP3 inflammasome mechanism. *Journal of Assisted Reproduction and Genetics* 40:1173–1185. DOI: 10.1007/s10815-023-02768-1.

X Z, W N, W Z, W N, C S, Y G, Z G. 2024. Multi-regulatory potency of USP1 on inflammasome components promotes pyroptosis in thyroid follicular cells and contributes to the progression of hashimoto’s thyroiditis. *Molecular Medicine (cambridge, Mass.)* 30:121. DOI: 10.1186/s10020-024-00885-w.

Zheng F, Ma L, Li X, Wang Z, Gao R, Peng C, Kang B, Wang Y, Luo T, Wu J, Yang Y, Gong L, Li Q, Yang S, Hu J. 2022. Neutrophil extracellular traps induce glomerular endothelial cell dysfunction and pyroptosis in diabetic kidney disease. *Diabetes* 71:2739–2750. DOI: 10.2337/db22-0153.
